# Smuggling Drugs into the Brain: An Overview of Ligands Targeting Transcytosis for Drug Delivery across the Blood–Brain Barrier

**DOI:** 10.3390/pharmaceutics6040557

**Published:** 2014-11-17

**Authors:** Julia V. Georgieva, Dick Hoekstra, Inge S. Zuhorn

**Affiliations:** Department of Cell Biology, University Medical Center Groningen, University of Groningen, A. Deusinglaan 1, 9713 AV Groningen, The Netherlands

**Keywords:** blood–brain barrier, targeted drug delivery, nanocarriers, transcytosis

## Abstract

The blood–brain barrier acts as a physical barrier that prevents free entry of blood-derived substances, including those intended for therapeutic applications. The development of molecular Trojan horses is a promising drug targeting technology that allows for non-invasive delivery of therapeutics into the brain. This concept relies on the application of natural or genetically engineered proteins or small peptides, capable of specifically ferrying a drug-payload that is either directly coupled or encapsulated in an appropriate nanocarrier, across the blood–brain barrier via receptor-mediated transcytosis. Specifically, in this process the nanocarrier–drug system (“Trojan horse complex”) is transported transcellularly across the brain endothelium, from the blood to the brain interface, essentially trailed by a native receptor. Naturally, only certain properties would favor a receptor to serve as a transporter for nanocarriers, coated with appropriate ligands. Here we briefly discuss brain microvascular endothelial receptors that have been explored until now, highlighting molecular features that govern the efficiency of nanocarrier-mediated drug delivery into the brain.

## 1. Introduction

Effective delivery of drugs across the blood–brain barrier (BBB) is a major limiting factor in successful therapy of brain diseases. The BBB is constituted by the specialized endothelium of the brain microvessels that preserves brain homeostasis by restricting the influx of a variety of compounds, including biomedicine, and yet enabling the supply of nutrients. Indeed, the potential treatment of many brain-related diseases is severely hampered because of the fact that numerous potential drug candidates are recognized as “brain-hostile”, thus precluding their penetration across the BBB into the brain. To overcome this problem, much research efforts are currently focused on the development and application of safe and efficient delivery devices, capable of promoting drug transport across the BBB. These efforts include the ability to achieve specific delivery by means of targeting to the BBB endothelium, followed by effective transport of the delivery device across the endothelial cell barrier and subsequent release of the drug at appropriate sites within the brain.

Here we will first briefly discuss the anatomical and molecular nature of the BBB. Obviously, such knowledge will be imperative for appreciating the need for development of state-of-the-art molecular devices to enable crossing of the barrier. We will subsequently discuss the presence of distinct receptors on the cell surface of vascular endothelial cells, capable of binding specific ligands and whose mechanism of internalization can be exploited for achieving targeted delivery and passage of a variety of nanodevices across the BBB.

## 2. Blood–Brain Barrier—Morphology and Function

### 2.1. The BBB as a Physical Barrier

Anatomically, the organization of the BBB is comprised of endothelial cells, basal lamina, astrocytes’ end-feet and pericytes. Endothelial cells of the BBB, in contrast to the systemic endothelium, are tightly connected to each other by means of tight junctions (TJ) and adherence junctions, and consequently lack fenestrations. Among others, proteins of the claudin family, *i.e.*, claudin 3, 5 and 12, occludin, and junction-adhesion molecules (JAMs), which are expressed at opposing cell surfaces, form the tight junctions by means of homophilic interactions. In this manner TJ strands “zip” adjacent endothelial cells and close the paracellular space for access of blood-borne molecules. The structure is further stabilized by ZO-1, -2 and -3, and MAGUK family proteins that link the TJ with the actin cytoskeleton. Signaling and regulatory proteins include multi-PDZ-protein 1 (MUPP1), the partitioning defective proteins 3 and 6 (PAR3/6), MAGI-1,-2,-3 (membrane-associated guanylate kinase inverted 1–3), ZO-1-associated nucleic acid-binding protein (ZONAB), afadin (AF6), and Regulator of G-protein signaling 5 (RGS5) [1]. Importantly, the presence of the tight junctions divides the plasma membrane of the vascular endothelial cells into two separate domains, that is the apical membrane, which faces the blood, and the basolateral membrane, which faces the brain tissue. The tight junctions preclude randomization of the surface components localized at each of these domains, thus warranting a specific composition of each domain. Basolaterally from the TJ, PECAM and VE-cadherin form the adherence junctions, which are linked to the cytoskeleton via desmoplakin and p120 catenin [1,2,3]. The structure is further supported by the basal lamina, a 30–40 nm thick matrix, composed of collagen type IV, heparin sulfate proteoglycans, fibonectin, laminin and other extracellular matrix proteins [4].

Multiple basal lamina proteins, matrix metalloproteases (MMPs) and their inhibitors, *i.e.*, the tissue inhibitor of metalloproteases (TIMPs), are involved in regulating the dynamics of the integrity of the BBB at physiological and inflammatory conditions [5]. In addition, molecular factors released from glial cells, such as glial-derived neurotrophic factor (GDNF), angiopoietin-1 [6] and angiotensin II [7] also contribute to the robustness of the integrity of the BBB. Furthermore pericytes regulate the developing BBB [8] and control the vascular permeability by restricting transcytosis, a transcellular transport process (see below) through PDGFR-β signaling [9] and regulation of Mfsd2a expression at the BBB [10]. Lastly, stability of the BBB is also provided by interactions with astrocytes, which cover more than 99% of the basal capillary membrane.

### 2.2. The BBB as a Functional Barrier

Apart from acting as a morphological barrier as such, the endothelial cell surface also contains molecular entities that further effectuate its barrier function. Thus, transporters belonging to the family of the ATP-binding cassette (ABC) transporters are expressed on the apical membrane surface of the BBB and their activity precludes entry and/or facilitates extrusion of many xenobiotics and endogenous compounds. In this manner, the functional activity of ABC transporters contributes to the barrier function of the vascular endothelium.

In an ATP-dependent manner the ABC-transporters actively extrude undesired compounds into the extracellular milieu. P-gp, MRP1 and BCRP are the most relevant ABC transporters at the BBB, while P-gp, consisting of two six transmembrane spanning domains and two ATP-binding sites, is the most active one. Common substrates for P-gp are cationic compounds or compounds with hydrogen bond acceptor groups [11], but also peptides [12]. Other efflux pumps expressed at the apical surface of the BBB endothelium are MRP4 and MRP5, whereas MRP4 is also present on the basolateral membrane.

Given the structural and functional tightness of the barrier, it is apparent that for purposes of drug delivery into the brain, development of sophisticated devices are needed, capable of passing the vascular endothelial cell lining. Potential mechanisms to be exploited are those based on paracellular entry (“between cells”) or via transcytosis (“through cells”). To appreciate the potency of these events we will therefore briefly describe the nature of such transport processes, occurring at (patho) physiological conditions.

## 3. Passive and Active Mechanisms of Transport across the BBB

Although preventing random influx of molecular compounds into the brain, the BBB does allow selective entry of nutrients and supply of regulatory factors into the brain. Thus the endothelial cells enable transport of molecules in a passive, gradient-driven manner or active, energy-dependent mode.

**Figure 1:**
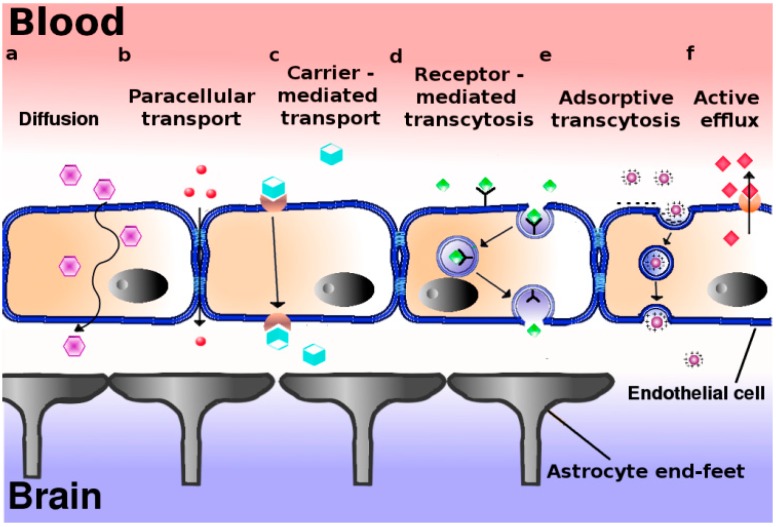
Proposed mechanisms of transport across the blood–brain barrier. The blood brain barrier consists in essence of a polarized layer of vascular endothelial cells, tightly attached to each other by means of tight junctions, and lined up by astrocytes. A variety of transcellular transport processes can be distinguished: (**a**) Diffusion, driven by a concentration gradient, mainly involving small hydrophobic molecules. This pathway represents the main entry route into the brain of current therapeutics; (**b**) Paracellular transport–limited to small water-soluble molecules; (**c**) Carrier-mediated transport, as occurs for e.g., glucose, amino acids, nucleosides, and therapeutics such as vinca alkaloids, azidothymidine *etc.*; (**d**) Receptor-mediated transcytosis for peptidic signaling and regulatory molecules (insulin, leptin, interleukins), nutrients (iron, LDL); (**e**) Adsorptive transcytosis, presumably relying on transport of positively charged cargo (serum proteins) in a non-specific manner; (**f**) Proton pump efflux transporters.

At physiological conditions, passive movement of water solutes along the aqueous route of the intercellular cleft between endothelial cells via small pores in the tight junctions, *i.e.*, the paracellular pathway, is negligible (Figure 1a). Since the exact molecular nature of paracellular transport is still largely obscure, and neurodegenerative and neuro-oncological diseases at the early stages of development are not necessarily accompanied by defects in BBB integrity, general exploitation of this pathway for transport of drug-loaded nanoparticles into the brain might be virtually negligible. As an alternative, transport via the endothelial cells *per se*, *i.e.*, by means of transcellular transport or transcytosis should thus be considered. Indeed, transport of small molecules through cells is a common event in polarized cells, including vascular endothelial cells (Figure 1b). Thus hydrophobic molecules, like many psychoactive drugs, with a size less than 500 Da, may diffuse transcellularly-provided they escape from MDR-mediated extrusion-to reach the brain parenchyma from the systemic circulation. However, transport of nutrients and building blocks is more tightly regulated and facilitated by specialized transporters (Figure 1c). For example, glucose uses the glucose transporter (GLUT1), while amino acids are transported by the large neutral amino acid transporter (LAT1). The latter transporter is also involved in transport of drugs like l-dopa, gabapentin, and baclofen [13]. For a detailed review of carrier-mediated transport at the BBB, see [14].

Whether these transporters, being primarily involved in molecular translocation of relatively small molecules, might similarly translocate larger sized nanoparticles into and out of the cell, appears less likely. Arguing that the brain depends on an extremely high glucose demand, *i.e.*, nearly 30% of the total body glucose consumption, liposomes prepared with glucose-modified cholesterol have been applied for brain-directed transport. These studies have shown a mild advantage in transporting coumarin-6 into the brain, over conventional liposomes. Although attempts have been undertaken to tie the GLUT1 transport system to transport of glucose-functionalized nanoparticles [15,16] the involvement of GLUT1 was not confirmed, or completely excluded in favor of passive cytoplasmic transfer [17]. Glucose-modified paclitaxel-loaded polymeric nanoparticles were successfully employed in the treatment of intracranial glioma in mice. Here, a role for GLUT1 in the transport of the nanoparticles across the BBB was confirmed [18].

Yet, a focus on receptors that internalize ligands by means of vesicular transport appears a more appropriate option since such intracellular transport vesicles can readily accommodate nanoparticles with a size of 100–200 nm. Indeed, transcellular transport in this manner (“transcytosis”) is a common phenomenon in polarized cells (Figure 1d,e).

Transcytosis represents a means of vectorial transport of cargo (including nanoparticles) between the apical and basolateral surface in polarized cells. It operates bidirectionally and three distinct steps in overall transcytotic transport can be distinguished: endocytosis of the cargo/nanoparticle at the plasma membrane (Box 1), followed by intracellular vesicular trafficking towards the opposite surface where exocytosis of the vesicular contents takes place. Transcytosis can rely on receptor-mediated and/or adsorptive, charge-dependent endocytic internalization events (Figure 1d,e). The adsorptive mode has been described to occur in particular for cationized serum albumin. Receptor-mediated transcytosis in brain endothelium may involve ligands like transferrin (iron), deltorphin, enkephalin, insulin, and LDL [5].

Here we will further discuss possibilities for specific targeting of nanoparticles into the brain relying on their specific processing along a transcytotic pathway rather than entry into an endocytosis-mediated degradation pathway (*cf*. Box 1).

**Box 1. Entry of molecules and particles into cells: Modes of internalization.**

**Figure 2:**
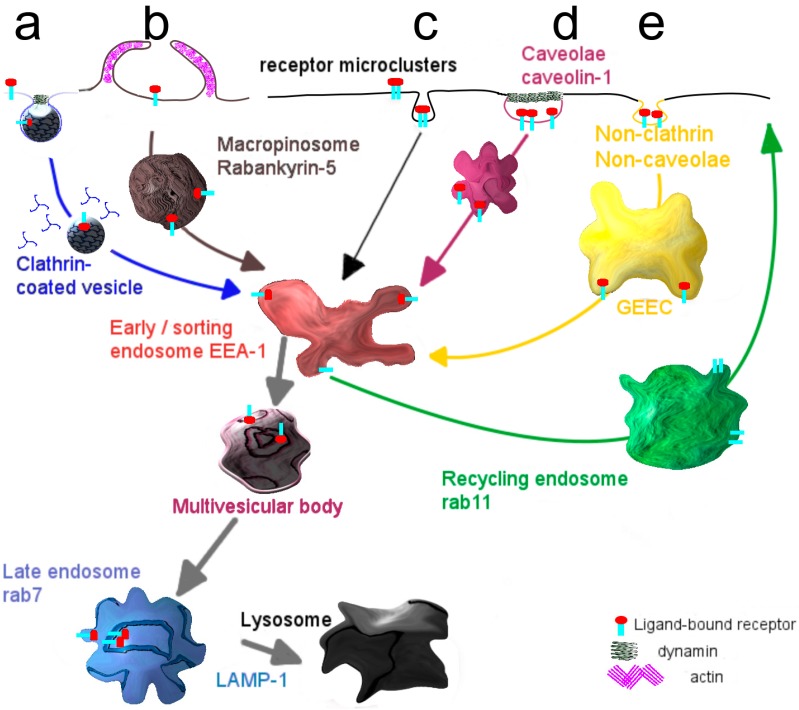
Mechanisms and transport pathways in endocytosis. (**a**) Adaptor molecules sense the binding of a ligand to its cognate receptor and cause the formation of a clathrin cage around the plasma membrane that subsequently pinches off as internal vesicle; (**b**) Caveolin-1, constitutively present at the inner leaflet of the plasma membrane, forms invaginated microdomains, known as caveolae, that mediate transport of receptor-ligand complexes; (**c**) A non-clathrin, non-caveolae dependent mechanism is mainly responsible for the uptake of glycosylphosphatidylinositol anchored proteins.

- Internalization via the endocytic mechanism is subdivided into clathrin-dependent, caveolin-dependent and clathrin- and caveolin-independent pathways (Figure 2). Clearly, the mode of cellular cargo entry is crucial for its eventual fate, as will be discussed below.
- Clathrin-mediated endocytosis involves the assembly of a clathrin coat at membrane regions enriched in receptor-ligand complexes, just underneath the plasma membrane, which subsequently triggers its inward budding. The small GTPase dynamin closes the neck of the invaginating membrane surface, leading to the formation of the clathrin-coated vesicle, which subsequently pinches off [19]. Shortly thereafter clathrin molecules dissociate from the vesicles, which then merge homotypically and/or with preexisting compartments that become enriched in EEA-1, a typical marker of early sorting endosomes [20]. From this compartment recycling may occur either directly to the plasma membrane, the so-called rapid recycling route [21,22] or via a recycling endosome, the slow recycling route [21]. Alternatively, early endosomes deliver their cargo to late endosomes, either via maturation [23] or by means of a vesicular transport mechanism [24] and eventually to lysosomes where its degradation may take place. Along the transition from early to late endosomes, the compartmental pH gradually drops [25].
- A better known but still poorly defined pathway capable of transferring cargo across the endothelial cell lining, constituting the blood-brain barrier, appears to rely on entry via caveolae [26,27]. As an advantage, lysosomal delivery as often occurs for entry along the clathrin-mediated pathway, may be avoided thus promoting secretory transport, rather than capture of cargo in a digestive compartment [28]. Accordingly, targeting specific receptors associated with caveolae may therefore well represent a far more appropriate strategy in facilitating transcellular transport, including that of targeted nanodevices. Caveolae are plasma membrane invaginations, commonly described as structures with a flask-like shape [29]. They are predominantly present at the surface of adipocytes [30], lung epithelium [31] and vascular endothelium [32,33]. A major constituent of caveolae is caveolin-1, which localizes at the inner leaflet of the plasma membrane [34]. Supported by the cavins-1, -2, -3 and -4, caveolae sustain their characteristic morphology, but gradually flatten [35]. Caveolae are considered platforms, where trafficking and signaling events take place [36,37,38].
- Stimulation of caveolar receptors triggers their internalization involving formation of a vesicular structure, named cavicle [39]. Cargo taken up by caveolae might be delivered to the Golgi apparatus or the endoplasmic reticulum, as reported for the cholera and shiga toxin B subunits in epithelium [40].
- A caveolar transcytotic route has been proposed to operate in endothelial cells of the BBB. Specifically, this pathway has been described for LDL particles which, prior to their release at the basolateral surface (brain side), localize at multivesicular bodies [41].
- The molecular organization of caveolae is strongly dependent on the presence of cholesterol, and agents that perturb the cholesterol content and organization, such as cyclodextrin and filipin, frustrate caveolae-mediated internalization [42,43]. The kinase inhibitor genistein is also known for its capacity to block caveolar uptake [44].

## 4. Nanocarriers and Active Targeting Systems (Ligands)

As depicted in Box 1, for the purpose of nanocarrier-mediated drug delivery into the brain, transcytosis from the blood (luminal side) into the tissue (abluminal side) seems to be the preferred pathway of crossing the BBB, as imposed by the vascular endothelium. Since caveolae have been proposed to be particularly active as transcytotic portal of entry [45], such a strategy would thus require caveolar targeting of the nanocarriers, implying coupling of appropriate ligands to these devices. The concept of coupling ligands directly to the therapeutic agent or to the surface of therapeutics-containing nanocarriers thus aims at (i) tissue specific delivery and (ii) improved penetration via transcytosis. As suggested by Pardridge [46], the Trojan horse constructed in this manner should contain a ligand that meets the necessary characteristics in order to serve as an effective tool in brain targeting. When coupled to the Trojan horse, the ligand should retain its targeting capacity towards a transcytotic receptor, as simple endocytosis might result in trafficking towards lysosomes and hence degradation. Thus, the targeted Trojan horse should bind with high affinity to the correct BBB receptor, and this affinity should be retained after fusion/conjugation with the therapeutic compound. Furthermore an effective internalization of the nanodevice is essential for achieving efficient brain uptake of the complex *in vivo* (>2% ID/gram brain tissue is required). Such uptake should of course result in a pharmacological effect, an effect that also should be warranted after intravenous administration of the complex. In the following we will briefly discuss a variety of ligands that have been used in optimizing brain delivery. Several of these have acquired a vector status of their own, involving their direct linkage to the drug rather than linkage to a drug-containing device. However, the use of nanocarriers for drug delivery to the brain may be preferred to prevent degradation of the drug during circulation and increase its concentration at the target site, the latter being of critical importance for reaching a therapeutic effect.

### 4.1. Transferrin

The best-known example of receptor-mediated endocytosis, operating in endothelial cells constituting the BBB, is the transport of iron-loaded transferrin (Tf). The holo-transferrin (iron-bound form) interacts with the transferrin receptor (TfR) at the (blood-facing) apical cell surface and releases its iron in early endosomes, triggered by the (mild acid) pH drop. The apoTf–receptor complex is subsequently recycled to the apical plasma membrane through rab11-positive compartments. The relative abundance of the TfR on the BBB endothelial cells, rationalizes the use of Tf–TfR system as a brain targeting system in a number of studies. Tf-conjugated solid lipid nanoparticles (SLNs) clearly increase the bioavailability of quinine dihydrochloride in total brain (parenchyma and microvessels) of rats compared to the administration of the free drug [47]. However, the particles remain associated with the microvessels, and the effect is therefore unlikely due to the transport of the drug–SLN complex into the brain. Moreover, a high plasma concentration of endogenous Tf balances the saturation of the receptor and discredits the use of Tf as an efficient targeting vector. Alternatively antibodies, with affinity for different epitopes on the TfR were therefore generated and investigated.

The radiolabeled anti-transferrin receptor monoclonal antibody OX26 was recovered in the brain of rats after intravenous injection, suggesting transcytosis across a functional BBB [48]. This observation subsequently accounted for the use of OX26 in many studies as a transport vector. Indeed, antisense oligonucleotides (ODNs) and peptide nucleic acids (PNAs) emerge as therapeutics. When PNA was conjugated to OX26, through biotin-streptavidin coupling, brain uptake, which is negligible for unconjugated drug, equals 0.08 ID%/g (% injected dose per gram tissue) after carotid artery perfusion. According to the authors this dose might be sufficient to elicit an effect with therapeutic significance, alluding to the brain recovery dose of injected morphine (0.1 ID%/g) [49]. Likewise, the mAb OX26 promotes the brain uptake of functionalized polymersomes (PO), suggesting retention of targeting properties when coupled to a nanoparticle [50]. The number of OX26 molecules exposed on the surface of a PO (100 nm) was optimal at 34 per particle, which yielded a brain recovery value of 0.136 ID%/g after i.v. injection in rats. Further increase in the OX26 concentration failed to produce higher brain uptake, possibly due to an increased liver accumulation.

As noted above, an important criterion to be met for effective targeting is the ability to accomplish a pharmacological effect, following delivery of the drug-containing device. Interestingly, such an effect was indeed obtained for the OX26-targeted polymersomes, loaded with the NC-1900 peptide, known to ameliorate spatial memory deficits. Thus, in scopolamine provoked rats, subcutaneous injection of the peptide *per se* and intravenous injection of its OX26 polymersomal formulation both improved the performance of the animals in water-maze tests. However, when encapsulated in OX26-conjugated polymersomes, a dose of NC-1900 lower than the free drug sufficed to produce a comparable effect, suggesting a more efficient brain delivery by means of polymersome-mediated transport [50]. Similarly, the effect of loperamide to produce analgesia was demonstrated. The drug is BBB impermeable and hence analgesic activity is measurable only after effective brain penetration. Tf-, OX26-, and RI7217 (mouse equivalent of OX26)-targeted albumin nanoparticles (160–185 nm) produced significant pain relief compared to mock-targeted (IgG2a) nanoparticles [51]. However, these antibodies lack the capacity to mediate actual crossing of the endothelial cell layer, as was determined *in vivo* using capillary depletion and morphological examination, resulting in capture of the antibodies and antibody–drug/nanocarrier conjugates within the brain vasculature [52,53,54]. A biphasic behavior of antibody–drug/nanocarrier conjugates with firstly, actual accumulation of the complex in endothelial blood capillaries, that has been documented, and secondly a passive release of the drug from the basal site might explain their efficacy in producing a pharmacological effect. Then, a low-affinity antibody would serve an improved delivery, as it will allow for easier release of the drug from the complex, once inside the cell [55]. In addition, carrier systems that will degrade within the brain endothelial cells may improve the subsequent transport of cargo further into the brain. It has also been postulated that novel ligands that prevent the dimerization of Tf receptors at the cell surface, thereby influencing the mode of internalization and subsequent intracellular trafficking, promote actual transcytosis [56]. Overall, these data support the concept that targeted brain delivery of nanodevices appears a realistic goal, and that the application of using an antibody against the transferrin receptor as brain vector, could serve that purpose.

### 4.2. Melanotransferrin

Melanotransferrin (p97) is a GPI-anchored protein that is highly expressed in melanomas. The protein, similar to Tf, is an iron-binding protein [57], and compared to Tf shows a 14-fold enrichment in the brain following injection of the recombinant form. Rather than the Tf-receptor, evidence supports a role of the low-density lipoprotein-receptor related protein (LRP) in the transport of p97 across the endothelium [58]. The targeting properties of p97 were demonstrated when covalently linked to paclitaxel or adriamycin. The total accumulation of the p97-drug complex in the brain reached a 10-fold higher level than that of the free drug. Furthermore, when linked to p97, the pharmacological activity of adriamycin was preserved and, when compared to non-targeted adriamycin, significantly decreased the progression of intracranial gliomas and mammary tumors [59]. After 24 h, p97-conjugates outperformed other, similarly designed vector-drug conjugates [49] and reached delivery levels of 1%–2% of the injected dose, which is equivalent to the ratio of brain to body weight. Hence, the p97 was thus claimed as the first carrier system to approximate this biological feature. Thus, melanotransferrin is preferentially transported from blood into brain tissue across the BBB, *i.e.*, in an apical to basolateral direction with a kinetic profile that reflects a low-affinity binding to a receptor with a high transport capacity. Moreover, in contrast to the plasma concentration of transferrin, the plasma concentration of endogenous p97 is relatively low, thus assuring ready access of melanotransferrin to available binding sites. Accordingly, relative to the TfR system, the melanotransferrin-receptor system emerges as a preferred targeting vector for drug transport into the brain.

### 4.3. Insulin

Insulin is transported to brain tissue from the circulation by means of a transcytotic mechanism, involving the insulin receptor present at the vascular endothelial cell surface [60]. The human anti-insulin receptor monoclonal antibody (HIRMAb) was shown to shuttle a TNFα decoy receptor (TNFR), which would counteract the effects of TNFα in inflamed brain regions, into the brain of Rhesus monkeys following i.v. administration. Although the detected values remain low, the concentration of the HIRMAb-TNFR fusion protein in the brain parenchyma was calculated as sufficient to neutralize the effect of TNFα in inflammatory conditions [61]. In the same study the inability of the fusion protein between TNFR and an antibody against the microvascular FcRn receptor (involved in the transcytosis of IgG) to cross the BBB, was explained by the predominant efflux of the FcRn receptor from the brain to the blood, *i.e.*, by its reverse transcytosis [62]. Although the HIRMAb was engineered to bind with high affinity to a different receptor epitope than the endogenous insulin, sequestration of the HIR from the plasma membrane by means of HIRMAB-drug conjugates would make it unavailable for its endogenous ligand–insulin, and *vice versa*. The competition between endogenous ligands and HIRMAb-modified drug conjugates for the same receptor may result in lack in efficacy of the nanoformulation and/or changes in receptor activity, of which the latter may have negative consequences for glucose metabolism [63].

### 4.4. LDL

The binding of low-density lipoproteins to the endothelial LDL-receptor (LDLR; [27]) or scavenger receptor-BI (SR-BI) initiates their transcytosis across the BBB. Remarkably, two endocytic pathways may be involved in the processing of the LDLR, which appears to depend on cell development. Thus, in non-differentiated, proliferating endothelial cells the LDLR is processed along the classical clathrin-mediated pathway (Box 1). LDL particles, when bound to the LDLR are taken up and delivered to late endosomes/lysosomes to meet the cell’s nutritional needs in terms of cholesterol supply. In this process, the receptor *per se* recycles back to the cell surface after dissociation of the ligand in early endosomes. However, in fully differentiated endothelial cells, constituting the BBB, the LDLR switches from a recycling to a transcytotic pathway. In this case, the receptor is captured by lateral diffusion in caveolae which, following their internalization, deliver intact LDL particles from the blood into brain tissue via intermediate multivesicular bodies (MVBs) (Box 1). Collectively, these data thus rationalize the use of LDLR as an appropriate transport system for drug delivery into the brain.

The first evidence to support this hypothesis comes from a study of Alyautdin *et al.* [64], which reported an analgesic effect after intravenous injection of dalargin (a hexapeptide analog of Leu-enkephalin, containing d-Ala in the second position and an additional *C*-terminal arginine), absorbed onto polysorbate 80-coated poly(butyl cyanoacrylate) (PBCA) nanoparticles. A series of follow-up studies [65,66,67] subsequently supported the enhanced drug delivery, as accomplished by using polysorbate 80-coated PBCA nanoparticles. Initially, the translocating effect across the endothelial cells was attributed to a general toxic effect of polysorbate 80 on the cells by causing a disruption of the tight junctions [68]. However, more recent data [69,70], in conjunction with the observation that serum apolipoproteins could adsorb on the surface of surfactant-coated nanoparticles [71] thereby promoting PBCA nanoparticles-endothelial cell interactions, support the currently prevailing hypothesis of receptor-mediated transcytotic transport of PBCA nanoparticles across BBB endothelial cells. Thus after intravenous injection, polysorbate 80 facilitates adsorption of the LDLR ligands, serum ApoB and/or ApoE onto the nanoparticles. As a consequence, the nanoparticles are targeted towards the LDL receptor on the brain capillary endothelial cells and are internalized, mimicking transcytosis of natural lipoproteins. Obviously, these observations subsequently raised an interest in surfactant-modified nanoformulations for brain delivery. Indeed, a nanosuspension of Amphotericin B prepared with Tween-80 (trade name of polysorbate 80) showed an enhanced adsorption of ApoE and ApoJ, and caused a moderate survival effect in *Balamuthia mandrillaris* encephalitis [72]. PBCA nanoparticles (35–40 nm) also proved to promote the CNS bioavailability of rivastigmine and tacrine as applied in the treatment of Alzheimer disease [73]. However, the PBCA nanoparticles also show several disadvantages, including a limited drug loading capacity, a low rate of *in vivo* biodegradation, and the release of toxic formaldehyde residues. Accordingly, physiologically better tolerable solid lipid nanoparticles (SLNs) have been suggested as suitable alternatives, relying on polysorbate 80 SLN preparations for further *in vitro*/*in vivo* studies [74]. Although polysorbate 80 is generally recognized as a safe emulsifier and included in many pharmaceuticals, care should be taken when a formulation is intended to be used for long-term parenteral application. For example, when treatment of a given disease will require repeated drug administration over periods of months, rather than a single shot, a higher toxicological burden caused by surfactant-nanoformulations might be a limiting factor, particularly in the treatment of chronic diseases.

Nevertheless, the concept of targeting the LDL-receptor to piggyback PBCA nanoparticles across the BBB, proved successful. Interestingly, essential for the targeting properties is not the surfactant itself, but rather, the apolipoproteins it attracts from the serum. Therefore, a variety of other apolipoprotein species were tested in order to verify their potency in targeting LDLR. Thus it has been shown that ApoE, covalently attached to human serum albumin nanoparticles (ApoE–HSA, size of approx. 250 nm), causes a relatively fast uptake of such particles (within 15–30 min) by brain capillary endothelial cells *in vivo* [75], and their processing by transcytosis was inferred from ultrastructural analyses. As a control, the authors demonstrated that pegylated nanoparticles (size of approx. 210 nm) do not associate with BBB endothelium, and consequently were not observed in brain parenchyma. Pegylation is a modification, usually carried out to prolong the circulation half-life of drugs and/or nanoparticles [76]. In this particular case, the control experiment may not have been an appropriate control given the rapid uptake of the non-pegylated particles, and hence questions the time span of the control experiment. Nevertheless, ApoE–HSA nanoparticles are transcellularly transported across the BBB and reach the brain intact, enabling brain delivery of drugs that usually cannot cross the BBB. The intracellular trafficking of ApoE–HSA was studied in an *in vitro* BBB model, *i.e.*, bEnd3 cells. After a 2 h incubation many ApoE–HSA particles localize in intracellular compartments that were similar in appearance to lysosomes. Considering some recent advances in the mechanism of LDL transcytosis [41], revealing LAMP-positive multivesicular bodies as an intermediate step in the process of transcytosis of LDL, the observed localization of the ApoE-HSA in lysosome-like compartments could thus reflect a very similar transcytotic trafficking route.

Comparable to Apo-E, other apolipoproteins, e.g., ApoA-I and ApoB-100, may also act as vectors for transport of loperamide-HSA nanoparticles into the brain [77]. ApoA-I is mainly associated with HDL particles, mediating internalization via the SR-BI, suggesting that SR-BI may function as an entry promoting receptor for ApoA-I–HSA particles. When scored by a fast antinociceptive response (15 min after injection), ApoE–HSA particles produce the highest effect, followed by ApoA-I–HSA particles, loaded with the same concentration of loperamide. Consistently, ApoA-I-HSA particles have been visualized in different brain regions [78].

The expression level of LDLR on the luminal surface of the brain capillary ECs is synchronized with the demand for brain lipid requirement, and strictly controlled by the underlying astrocytes [79]. Thus when their cholesterol level is limited, astrocytes will transmit signals that lead to an up-regulation of luminal endothelial LDLR. A limited expression of LDLR will consequently result in a saturable transport capacity, thereby potentially limiting its use in brain delivery of cargo-containing nanoparticles. This implicates that drug delivery efficiency will likely be influenced by body rhythms, such as eating rhythm. Moreover, similarly to the transferrin receptor, LDLR is also expressed in various other tissues with the highest functionality in hepatocytes, implying a lack of brain capillary endothelium specificity.

### 4.5. Angiopeps

Low-density lipoprotein receptor-related protein (LRP) 1 (alpha-2-macroglobulin receptor) and 2 (Megalin) are other members of the LDL receptor family that are widely studied for their ability to transport multiple ligands across the BBB. Well-characterized are the LRP-dependent endothelial transport of lactoferrin [80], receptor-associated protein (RAP) [81], tissue plasminogen activator (tPA) [82], and secreted amyloid precursor protein (APP) [83,84]. Particularly in the latter case, when the Kunitz protease inhibitor (KPI) fragment was sequestered from the secreted APP, the LRP-dependent uptake and consequent degradation of APP was reduced [84]. Additionally, the transcytosis rate of ^125^I-aprotinin, another example of a KPI domain containing protein, across endothelium in an *in vitro* BBB coculture model or *in situ* brain perfusion, was higher when compared to holo-transferrin, used here as positive control. These observations were taken to suggest the involvement of the Kunitz domain in ligand recognition and subsequent LRP-mediated endocytosis. Accordingly, these findings rationalized the development of designing KPI-based peptides as vectors for brain delivery, operating through the LRP transport system [85]. Alignment of the amino acid sequence of aprotinin with that of bikunin, amyloid β A4 protein precursor, and the Kunitz inhibitor-1 precursor (all LRP ligands) led to the design of 96 peptides, commonly referred to as angiopeps. After *in vitro* and *in vivo* postselection, a peptide, designated as Angiopep-2 showed the highest transcytotic potential, and was used for further studies. The hypothetical role of LRP1 as a potential receptor for Angiopep-2 was examined by means of competitive inhibition experiments and colocalization studies, as carried out by fluorescence microscopy. Apical to basal transport of α2-macroglobulin (LRP1 specific ligand) across bovine brain capillary endothelial cells (BBCEC) was partially inhibited in the presence of Angiopep-2, and *vice versa* [86]. Furthermore, fluorescently-labeled Angiopep-2 colocalized in BBCEC to a certain extent with LRP1 in intracellular vesicles, pointing towards at least a partial LRP1-mediated transport of Angiopep-2. The potency of Angiopep-2 in brain delivery became evident when used as a conjugate, consisting of three molecules of paclitaxel, covalently attached to one Angiopep-2, also known as ANG1005. Paclitaxel preserves its cytotoxic effect in this form, and after intracerebral implantation of primary or metastatic carcinomas, administration by intraperitoneal injection of ANG1005 increases the median survival rate and prolongs the life span of mice, compared to vehicle, *i.e.*, Angiopep-2 without paclitaxel treated animals [87]. Currently, ANG1005 is undergoing phase I/II clinical studies [88,89].

However, the potency of Angiopep-2 as a brain delivery vector is less pronounced when applied in conjunction with nanoparticle-mediated delivery. Thus when Angiopep-2 was grafted onto the surface of liposomes, the recovery of the targeted particles in the brain was negligible, and even less than that of liposomes grafted with RI7217 (the mouse analog of the transferrin antibody OX26, discussed above) [90]. Accordingly, these data would suggest that the transport pathway and/or transport efficiency of Angiopep-2 as target entity may very much depend on the attached cargo (drug *versus* nanoparticle). It is finally interesting to note that in BBB endothelial cells, newly synthesized LRP1 is sorted to the basolateral surface (*i.e.*, facing brain tissue) [91]. However, since LRP1 apparently operates as transporter from the basolateral to apical surface in endothelial cells [83], these observations would thus imply that the transient appearance of LRP1 at the apical, *i.e.*, blood facing surface, suffices to allow for an interaction with ligands like Angiopep-2 thus allowing in this manner brain directed transport via the opposite transcytotic pathway.

### 4.6. Leptin

Leptin is a regulator of body weight by controlling the food intake. Post alimentary, leptin is secreted from the cells in the gastrointestinal tract, and is subsequently transported by the system circulation to the brain endothelium. A specific receptor ObR on the luminal side of the brain capillary ensures leptin receptor-mediated transcytosis and its translocation to the brain stem to relay the satiety signal. Hence, these observations would support the potential of the ObR receptor to serve as a transport system in brain delivery. Liposomes with a size of approx. 180 nm, and decorated with a leptin-derived peptide showed a significantly enhanced uptake in brain endothelium, compared to liposomes, devoid of the leptin-peptide [92]. The internalization was sensitive to amiloride, but not filipin III or chlorpromazine, interfering with caveolae- and clathrin-mediated endocytosis, respectively. These data would thus suggest that these latter pathways as mechanisms for cellular entry of the leptin-targeted liposomes can be excluded. Rather, sensitivity toward amiloride would favor macropinocytosis as the mechanism of entry, and this pathway indeed has been linked to transcytosis in epithelia [93,94]. Of further interest, the tissue distribution of the ObR receptor is restricted, with a high expression at the BBB endothelial cells [95], while the receptor is occupied only after meals. These features are of particular interest and merit further investigations as to the development of the leptin–ObR system as a potential target in brain delivery. Notably, obesity in humans is thought to be caused by an impaired transport of leptin across the blood–brain barrier (BBB) [96], making leptin-targeted drugs potentially inappropriate for use in obese individuals.

### 4.7. Thiamine

Thiamine or vitamine B1 is a water-soluble nutrient with many physiological implications. At the BBB, the expression of the thiamine transporter facilitates its transport into the brain. The transport process of thiamine-functionalized nanoparticles has been investigated by Lockman *et al.* [97]. Thus, (3H) thiamine was exposed on the surface of solid nanoparticles (67 ± 8.2 nm), prepared from oil-in-water microemulsion, via incorporation of a distearoyl phosphatidylethanolamine (DSPE) anchor. Detailed kinetic profiling of the thiamine-tagged nanoparticles, after *in situ* rat brain perfusion, revealed a passive brain permeation of the particles, following an association with the BBB thiamine carrier. However, after systemic injection, the brain targeting specificity of thiamine-tagged nanoparticles is lost. Likely, similar to other targeting vectors, thiamine-coated nanoparticles might compete for binding sites with endogenous thiamine or associate with thiamine transporters, localized at other tissues. In summary, the current status and insight concerning the efficiency of transport across and expression of the thiamine receptor on endothelial cells, does not seem to justify high expectations concerning its exploitation as a suitable target for brain-directed delivery of drugs.

### 4.8. Glutathione

Glutathione, a natural anti-oxidant, is found at high levels in the brain, and its receptor is abundantly expressed at the blood–brain barrier. Hence, liposomes coated with glutathione-conjugated PEG (G-Technology^®^) proved to mediate safe targeting and enhanced delivery of drugs into the brain [98,99,100]. Currently, clinical trials with patients with brain metastasis, originating from breast cancer, are ongoing with the lead product 2B3-101, which is doxorubicin formulated with the same technology.

### 4.9. Synthetic Opioid Peptides

Enkephalins are neuroactive peptides that are transported bidirectionally across the BBB in a saturable and nonsaturable mode. The saturable mode likely arises from a specialized Peptide Transport System(s) (PTS; [101]). The PTS1 operates in a brain-to-blood direction and is responsible for the efficient clearance of enkephalins from the brain [102]. The opposite transport-brain influx-of enkephalins, although documented after *in situ* perfusion [103], is not sufficient to account for therapeutically meaningful amounts for systemic use [104]. Lipidation [105] or glycosylation compensates for the negative net influx, as demonstrated with peripherally administered glycoenkephalins that show antinociceptive activity comparable to that of morphine [106]. An effect that was correlated to glycoenkephalins enhanced blood-to-brain transport across the BBB and bioavailability. In subsequent work, the nature of brain influx of glyconeuropeptides was attributed to their amphipathic structure, and adsorptive endocytosis was proposed as main entry in the BBB endothelium [107]. Based on these findings and in an attempt to simulate similar transport mechanism across the BBB, a short seven amino acids peptide sequence was proposed to suffice for brain delivery [108]. The peptide, derived from the synthetic opioid peptide MMP-2200, which is an enkephalin analog, was deprived from “unnecessary” pharmacological activity and glycosylated at the *C*-terminal serine with sugar residues (d-glucose, d-galactose, d-xylose or d-lactose). Copolymer nanoparticles (200 nm) of poly (d,l-lactide-*co*-glycolide) (PLGA), assembled from peptide-modified PLGA copolymer, were administered by *in situ* perfusion. Confocal images of brain slices, obtained after perfusion, showed localization of the nanoparticles in the cerebral parenchyma for the glucose, galactose and the lactose variants, with higher accumulation of the glucose variant. Further, nanoparticles with the glucose-functionalized peptide were shown to accumulate in brain tissue, also after systemic administration. Proof of principle for therapeutic activity was obtained in several studies [109,110]. PLGA-nanoparticles, functionalized with the same glucose-bound peptide, were loaded with the model drug loperamide. The highly effective transport of loperamide (~13% of the injected dose) across the BBB following i.v. administration of the nanoparticle formulation resulted in a pharmacological effect. Moreover, total brain biodistribution studies, revealing that ~15% of the injected dose of the (fluorescently labeled) nanoparticles were transported into the brain, suggest the transport of drug-nanoparticle conjugates across the BBB. The macropinocytosis-like mechanism, which appears to depend on the Biousian structure of the peptide, is likely the mechanism by which these nanoparticles translocate across the BBB [111].

### 4.10. RVG Peptide

Rabies virus is a pathogen with a high neurotropism *in vivo*. The rabies virus glycoprotein (RVG) specifically interacts with the *N*-acetylcholine receptor on neuronal membranes, which mediates virus entry. A modified RVG peptide with 9 arginine residues that binds siRNA, was designed as a vector for transvascular delivery [112]. After intravenous injection into mice, the FITC-labeled siRNA, attached to the RVG-9R targeting entity, was detected in the brain, but not in spleen or liver. Functionality was demonstrated with anti-GFP siRNA, following intravenous injection. This construct silenced the GFP brain expression in GFP transgenic mice, with no significant effect on the GFP levels in spleen and liver. The therapeutic potential was further explored in mice with fatal flaviviral encephalitis. RVG-9R bound to antiviral siRNA rescued 80% of the infected animals. The acetylcholine receptor (AchR) is highly abundant in the CNS, including brain capillary endothelial cells. Binding of RVG to the alfa7 subunit of AchR likely promotes receptor-mediated transcytosis of RVG-9R–siRNA in brain microvessels. Hence, this vector emerges as a very promising tool when acetylcholinergic neurons are the desired target, as in the case of most senile dementias. However, RVG-9R–siRNA elicits IL-6 mediated immune responses. This effect was neutralized when employing exosomes, produced by self-derived dendritic cells [113], engineered to express Lamp2b, an exosomal membrane protein, fused to the RVG peptide, loaded with siRNA. The therapeutic potential of exosome-mediated siRNA delivery was demonstrated by a significant decrease in beta-secretase 1(*BACE1*) mRNA (60%) and protein (62%) levels in wild-type mice [113].

### 4.11. Tetanus Toxin, Tet1 and G23

Retrograde transport from distal peripheral axonal projections to the cell body, and transsynaptic spreading is a remarkable route used by the tetanus toxin (TeNT) to invade the CNS. Like other bacterial toxins, TeNT has two distinct functional peptide fragments. The *C*-terminal heavy chain fragment (TTC) is nontoxic, and piggybacks the light chain, which expresses pathogenic enzymatic properties. TTC *per se* displays a high affinity for GT1b, the receptor for TeNT, and is also efficiently transported along axons [114]. Indeed, targeting of fusion proteins containing the TTC fragment is readily accomplished. Thus after intramuscular injection of superoxide dismutase SOD–TTC [115] or β-gal–TTC fusion proteins [116] into the mouse tongue, the hybrid proteins follow the TTC retrograde route and are detected in higher order brain motor neurons. Also intraperitoneal injection of TTC protein hybrids into mice results in an enhanced CNS uptake [117]. Inspired by these observations, TTC has also been coupled to (d,l-lactide-*co*-glycolide) PLGA–PEG nanoparticles (size of approx. 250 nm) in order to accomplish transfer of the targeted nanoparticles to the brain parenchyma via the same transport pathway as the TTC fusion proteins [118]. As determined by flow cytometry a six-fold increase in binding of TTC-tagged nanoparticles was observed when binding to N18-RE-105 neuroblastoma cells was compared to that of HepG2 liver cells, thus reflecting the neuronal tropism of TTC. However, although such *in vitro* data are of interest, it remains to be determined whether *in vivo* these nanoparticles might reach with a similar effectiveness the CNS. Retrograde axonal transport of virus particles, which are similar in size as the applied nanoparticles is well documented [119]; it is therefore not *a priori* unlikely that TTC-nanoparticle transport may occur in a similar manner. Thus, TTC emerges as a highly potent vector in brain delivery. However, due to mass vaccination, its immunogenicity remains a serious concern. A potential solution to this problem has been provided by a phage library approach, biopanned on GT1b [120] in search of peptides displaying a binding efficiency and subsequent cellular processing, analogous to that of TTC. The identified peptide Tet1 fulfilled the required criteria. Importantly, Tet1 is similarly transported in a retrograde mode [121]. More recently, screening a peptide phage library against the ganglioside GM1, which is ubiquitously expressed in caveolae at the apical surface of vascular endothelial cells, we identified a peptide, defined as G23, with a sequence identical to Tet1 [122]. Detailed studies revealed that G23 effectively targets a transcytotic pathway in human brain capillary endothelial cells, and mediates efficient transport of G23-coated polymersomes across an *in vitro* BBB model [122]. Moreover, these studies also provided evidence for *in vivo* transfer across the BBB of intact peptide-targeted drug delivery vehicles into brain parenchyma, following intracarotid artery injection in mice. Further work confirmed that although specifically selected for GM1 as its targeting receptor, G23, like Tet1, also binds to the structurally similar ganglioside GT1b, implying that both gangliosides may play a role in transcytotic transport of G23-coupled polymersomes across the BBB. These promising observations warrant further investigations, in particular with regard to the design of G23-coated carriers that effectively mediate the actual delivery of drugs into the brain, *i.e.*, the release of carrier contents, preferably in a programmable manner.

### 4.12. Diphteria Toxin

The non-toxic analog of the diphteria toxin (DT), CRM197, might be advantageous as carrier system because its receptor HB–EGF (membrane-bound precursor of heparin-binding epidermal growth factor) is specifically expressed on the BBB endothelium, neurons and glia, while the expression is up-regulated in disease-related conditions. Furthermore, the receptor lacks endogenous ligands. HRP-conjugates of CRM197, Tf or BSA were compared as targeting vectors in an *in vitro* co-culture of bovine BCEC with rat astrocytes [123,124]. CRM197 promoted the translocation of HRP at 37 °C, whereas the process was blocked at 4 °C, suggesting an energy-dependent and presumably receptor-mediated uptake and transport. Histological examinations of saline-perfused guinea pig brains, after bolus injection of CRM197-HRP or Tf-HRP, suggests a preferential extravasation and accumulation of the CRM197-conjugated molecule in the brain. Whether the CRM197 vector is able to trigger effective transcytosis, *i.e.*, as effective as its toxic counterpart DT, remains to be determined.

### 4.13. TAT peptide

**Figure 3:**
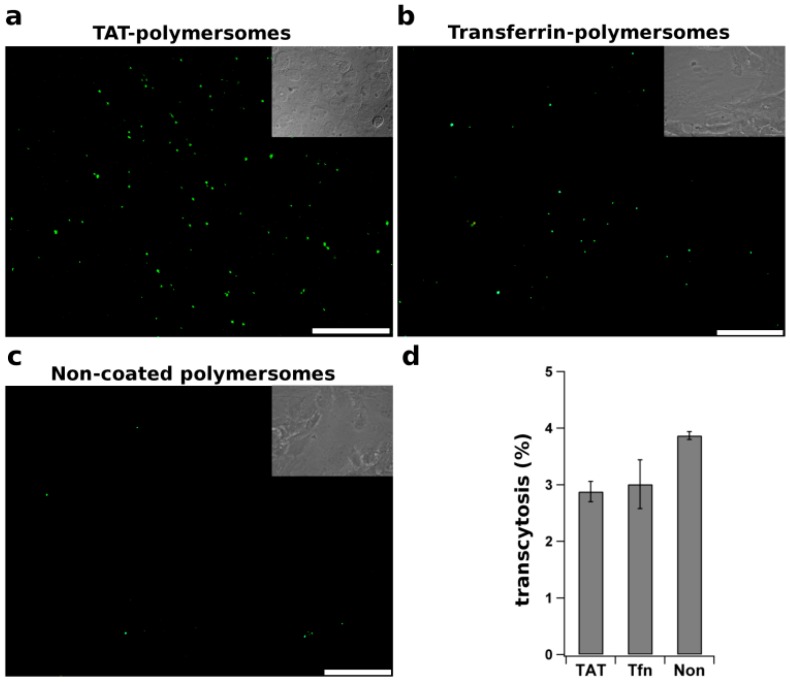
Internalization and transcytosis of functionalized polymeric nanoparticles. TAT peptide and transferrin were covalently attached to polymersomes and incubated for 18 h at 37 °C with polarized filter-grown endothelial hCMEC/D3 cells, a convenient *in vitro* model for the BBB (see [132]). After extensive washing to remove non-bound particles, the cells were examined by fluorescence microscopy to visualize the intracellular distribution of (**a**) TAT-, Transferrin (Tf)- and non-coated polymersomes, as indicated (Scale bar: 20 µm); Alternatively (**b**), after 18 h of incubation at 37 °C the fractions of polymersomes in the apical and basolateral medium were measured and the fraction of polymersomes, transported by transcytosis across the endothelial cells, was calculated.

Cell penetrating peptides (CPPs), *i.e.*, short cationic peptides, are able to transduce cargo across membranes. Through non-specific electrostatic interactions, CPPs adsorb strongly to the negatively charged cell surface [125]. Although the mechanism of CPPs translocation is still debated, involving issues, such as the formation of transient pores or inverted micelles, the conjugation of CPPs to cargo is thought to promote the rate and extent of cellular uptake. As an example of a CPP, the TAT (transduction domain of human immunodeficiency virus type-1 (HIV-1)) has been extensively studied. After intraperitoneal injection, the TAT peptide considerably increases the ubiquitous tissue penetration of a TAT–β-galactosidase conjugate beyond the capillaries, including the brain [126]. Based on these observation CPPs have been suggested to potentially act as vectors for enhanced brain delivery [127]. The suggestion was further investigated in a study with TAT-functionalized quantum dots (Qdots) for brain imaging. After intracarotid artery administration, the Qdots signal is detected in the brain parenchyma, although a very high accumulation of the particles was seen in arteries [128]. In contrast, in an *in vitro* study using a similar approach in which the peptide was linked to the cargo, GFP–TAT conjugates were shown to transmigrate through an intact endothelial monolayer, but to an extent very similar as observed for GFP alone [129]. These results thus question the role of TAT in mediating enhanced transport across the BBB. However, there appears to be no consensus as other data have suggested that the TAT peptide is of interest for nanoparticle-mediated drug delivery. In a study in which TAT-conjugated poly (l-lactide) nanoparticles were employed, loaded with the protease inhibitor ritonavir, prolonged sustained release of the drug in brain parenchyma was reported [130]. However, both TAT-conjugated nanoparticles and those devoid of TAT localized abundantly in the ventricles. Accordingly, it is also possible that penetration of released ritonavir may have occurred via cerebrospinal fluid into the brain, rather than via direct delivery involving nanoparticle transport across the BBB. In another study, self-assembled micelles prepared from TAT-PEG-b-cholesterol, labeled with Qdots, were detected 4 h after i.v. injection in rat hippocampus [131]. Whether the TAT–PEG-*b*-cholesterol micelles remain in the endothelial cells or do acquire access to brain cells is difficult to determine, without specific labeling. Indeed, data obtained in our laboratory provides evidence for a dramatic increase in association of TAT-coated polymersomes, relative to non-coated control polymersomes, to hCMEC/D3 BBB cells, in agreement with the afore mentioned studies (Figure 3a,c).

The effect, however, did not correlate with an increased transcytosis rate across the BBB endothelium *in vitro* (Figure 3b), thus questioning the potential of TAT as an effective vector for nanoparticulate brain delivery. Additionally, enhanced ubiquitous tissue accumulation of the cargo-ligand unit remains an essential obstacle of CPPs usability, as organ specificity was not demonstrated thus far.

## 5. Concluding Remarks

Here, we have discussed the rationale behind the selection of certain receptors, together with the pros and cons of their usability for brain drug delivery. An increasing number of studies on targeted delivery supports the initial hypothesis on a Trojan horse strategy: an appropriate receptor may ferry ligand-coupled cargo/drugs across the BBB. While direct comparison of the listed studies is precluded because of the different protocols used to assess the therapeutic outcome, clearly few vectors are emerging that may be considered as a successful “new generation” of targets. Nevertheless, the glutathione technology, developed for nanoparticle delivery, and the ANG1005 for direct drug-ligand delivery, already entered the stage of clinical evaluation for treatment of brain tumors. Also the tetanus toxin (TTC) and/or its functional competitor (Tet1/G23) and the RVG-9R peptide warrant special attention as very efficient vectors for drug delivery from the system circulation into the brain. The important advantage is that these vectors also hold natural neurotropism with specificity towards certain neuron subpopulations. Tet1 was developed to attack motor neurons and addresses unmet needs in treatment of amyotrophic lateral sclerosis. Similarly, targeting of acetyl cholinergic neurons with RVG-9R-acetylcholinesterase inhibitors would allow for determination of an optimal dosage and hence reduced adverse effects in symptomatic treatment of age-related memory impairments or any neuropathology with disturbed acetylcholine levels.

While researchers are still in search of the ideal vector, gathering more insight into the nature of transcellular transport, combined with identifying novel BBB specific receptors may pave future pathways in crossing the blood–brain barrier. Here, improvement of the current *in vitro* models of the BBB may further improve the predictive value of *in vitro* experiments, Cocultures of brain endothelial cells (ECs) with pericytes and astrocytes, thereby reconstituting the properties of the neurovascular unit, may reflect the *in vivo* properties of the BBB better than monocultures of brain ECs. Moreover, future research may benefit from imaging approaches using drug–carrier conjugates in which the drug and the carrier each carry their own label, allowing to measure the presence of intact conjugates as well as free drugs. This will help to determine the amount of free drug in the brain that is needed to exert a therapeutic effect, as well as provide information on the involvement of nanocarrier transcytosis across the BBB, and is therefore of great value for assessing the significance of the Trojan horse strategy for drug delivery into the brain.
